# Improved quality monitoring of multi-center acupuncture clinical trials in China

**DOI:** 10.1186/1745-6215-10-123

**Published:** 2009-12-27

**Authors:** Ling Zhao, Fan-rong Liang, Ying Li, Fu-wen Zhang, Hui Zheng, Xi Wu

**Affiliations:** 1 College of Acupuncture and Massage, Chengdu University of TCM, Chengdu, Sichuan 610075, China; 2 College of Clinical Medicine, Chengdu University of TCM, Chengdu, Sichuan 610075, China

## Abstract

**Background:**

In 2007, the Chinese Science Division of the State Administration of Traditional Chinese Medicine(TCM) convened a special conference to discuss quality control for TCM clinical research. Control and assurance standards were established to guarantee the quality of clinical research. This paper provides practical guidelines for implementing strict and reproducible quality control for acupuncture randomized controlled trials (RCTs).

**Methods:**

A standard quality control program (QCP) was established to monitor the quality of acupuncture trials. Case report forms were designed; qualified investigators, study personnel and data management personnel were trained. Monitors, who were directly appointed by the project leader, completed the quality control programs. They guaranteed data accuracy and prevented or detected protocol violations. Clinical centers and clinicians were audited, the randomization system of the centers was inspected, and the treatment processes were audited as well. In addition, the case report forms were reviewed for completeness and internal consistency, the eligibility and validity of the patients in the study was verified, and data was monitored for compliance and accuracy.

**Results and discussion:**

The monitors complete their reports and submit it to quality assurance and the sponsors. Recommendations and suggestions are made for improving performance. By holding regular meetings to discuss improvements in monitoring standards, the monitors can improve quality and efficiency.

**Conclusions:**

Supplementing and improving the existed guidelines for quality monitoring will ensure that large multi-centre acupuncture clinical trials will be considered as valid and scientifically stringent as pharmaceutical clinical trials. It will also develop academic excellence and further promote the international recognition of acupuncture.

## Introduction

Acupuncture is a typical traditional medicine that has been widely used in clinical practice for thousands of years in China. Because of its multi-advantages, acupuncture has been gradually obtained acceptance as an alternative or complementary treatment for health care in many western countries. In 1997, at a Consensus Conference sponsored by the National Institutes of Health, acupuncture was recognized as an effective treatment for chronic pain syndromes and for postoperative and chemotherapy-induced nausea and vomiting. Acupuncture has also received recognition as a beneficial adjunct for treating drug addiction, stroke rehabilitation and asthma[1]. In the UK, acupuncture is widely used in both private and NHS practice. In surveys of the use of complementary medicine, acupuncture is consistently cited among the most commonly used[2].

At present, the systematic review of evidence-based medicine (EBM) and large sample randomized controlled trial (RCT) are considered as the most reliable evidence which guides the clinical practice. The initial concept of EBM was defined as the "conscientious, explicit and judicious use of the available evidence in making decision about individual patient care"[3]. Currently, the integration of best external clinical evidence available from systematic research with individual clinical expertise and patient preferences is the standard in practice[4]. In assessing the effectiveness of treatment modalities involving human subjects, the RCT design is generally considered as the gold standard method with the least spurious causality and bias [5].

Recent years, many RCTs using acupuncture were carried out in Germany, USA, England and other western countries [6-8]. In China, the multi-center acupuncture studies with large sample of RCTs have generated a considerable amount of attention. Both National Key Basic Research Program (*973 Program*) and the "11th Five-Year Plan National Key Technology R & D" were designed as the multi-center randomized controlled trials, which were supported financially by China's Ministry of Science and Technology.

In order to complete multi-center acupuncture clinical trials with high quality, the Science Division of the State Administration of TCM of China convened a special conference about quality control on clinical research of TCM in 2007. The conference established a series of control and assurance standards to guarantee the quality of clinical research. We recognize the quality controls that already existed were not adequate for acupuncture clinical trials. The reporting quality of RCTs published in the five leading Chinese medical journals is low[9]. Based on the STRICTA checklist, the assessment of RCTs published in Chinese Acupuncture and Moxibustion indicates that the methodological quality was lower than the international standard, especially on acupuncturist's qualification, adverse reaction, blind method, flow of participants in the trial and follow-up, et al[10]. Poor reporting of RCTs give more urgency to improve quality monitoring of multi-center acupuncture clinical trials in China.

This paper provides practical guidelines for implementing strict and reproducible quality control for acupuncture RCTs. The clinical trials reported in this article are financed by the "*973program*" and "11th Five-Year Plan" from National Key Technology R & D program of China respectively, and both programs are registered with their own identifier (NCT00599586, NCT00608660) by Clinical Trials.gov in the USA. These trials are performed according to the principles of the Declaration of Helsinki (Version Edinburgh 2000). And the trial protocols have been approved by local institutional review board and ethics committee. Chengdu University of TCM takes charge of these studies. The first part of clinical research of "*973program*" (Grant No.2006CB504501) has published the initial results [11]. The second part of our project is undergoing in China and its study protocol has published in TRIALS [12].

## Methods

The practice of quality monitoring of these acupuncture trials focuses on making the standard quality control program (QCP). This QCP includes the design of case report form (CRF), the training of qualified investigators, study personnel and data management. All of quality control programs are completed by monitors who are directly appointed by the project leader. The duty of these monitors is to guarantee data accuracy and to prevent or detect protocol violations such as enrollment of noneligible patients or assignment of improper treatment and so on. Therefore, the monitors should satisfy the following requirements: (1) having medical background, mastering the strict training of GCP standard, and having abundant experience of clinical trials; (2) being familiar with protocol of the trial and standard operating procedures (SOPs); (3) monitoring the whole procedures of data management, including filling in the Case Report Forms(CRFs) and electro-CRFs as well as modification error; (4) having adequate time to do the monitoring work periodically according to the QCP.

### Preparation work on quality monitoring

Monitors should first understand and control the monitoring program. This scheme usually includes: (1) deciding the number of monitors in each clinical center, according to the number of centers; (2) planning the supervision speed in terms of clinical trial progress; (3) adjusting the inspection frequency based on the trial schedule.

At the same time, the monitors should draw up the monitoring assessment forms (see Additional File 1) which are developed from the GCP regulations, and these forms are designed according to the characteristics of acupuncture clinical trials. Questions are not rated per se; each assessment question has one of the possible responses: yes, or no, and some questions are provided with brief descriptions as well.

Implementation is the core aspect of the monitoring procedure, which includes the following steps:(1) reading the study summaries, feedback comments and on-the-spot suggestions from each clinical center before the inspections;(2) maintaining a good communication with the director of each centre, clinicians, patients and laboratory examiners, to be informed of every concrete flow of procedure and the progress;(3)taking notes about the problems, flaws, and on-the-spot photos;(4)summarizing the monitoring results and evaluate the integrity and credibility of clinical data collections.

### The practice of quality monitoring

#### 1. Audit the preparation work in every clinical center

We check every clinical center which participates in these clinical trials to see how much they value the project and check the use and management of clinical diagnosis equipments, acupuncture appliances and the laboratory examination instruments to see whether these instruments are in line with the requirements of SOPs. Meanwhile, the monitors should examine if there is any change to the clinical investigators, and if all of them are well trained or have passed the exam on implementation scheme which is organized by the sponsor.

#### 2. Inspect the master degree of procedures

The clinicians play an important role and participate in most of steps during the implementation of the studies. Their duties are not exactly the same in each period of trial, such as recruiting, enrolling, screening, randomized allocation, treating patients and follow-up. Therefore, it is indispensable to ensure that the clinicians grasp the procedures systematically.

#### (1) Inspecting the usage of center-randomization system

Monitors inspect the usage of center-randomization system and ask if the clinicians have confirmed the case histories and laboratory tests that meet inclusion criteria. They also check whether the clinicians grasp the procedure of applying center-randomization and whether they can remember the English alphabets, which represent specific divisions in the feedback messages through telephone, mobile phone, mobile-phone message, or website.

#### (2) Auditing the process of treatment

It is necessary to analyze the process of treatment and operations. Firstly, monitors check whether the diagnosis, disease stages and classifications conform to the inclusion criterias. We use the patient's medical records, the corresponding conclusions of laboratory examinations, as well as the records of patients with symptoms, such as the headache diaries of attack frequency, attack duration, simultaneous phenomenon to determine the certainty of basic information of illness.

Secondly, we inspect whether or not the course of the treatment strictly corresponds to the randomized implementation of the corresponding treatment measures including the selection and location of acupoints or non-acupoints, acupuncture manipulation, the operation of electro-acupuncture, SOPs adherence, etc. All procedures should be recorded by taking pictures.

Thirdly, we pay special attention to the physicians' explanations on whether we should follow the clinical research requirements and the GCP regulation during our communication with the subjects. Due to the unique nature of acupuncture operations, the double-blind cannot be adopted between subjects and physicians. However, the clinicians cannot assess the efficacy of treatment on subjects to avoid misleading the patients, and therefore, cause potential impact on the compliance and outcome measurement. For example, patients are randomly divided and may be acceptable to the treatment of non-acupoints or acupoints. The clinicians should not imply which method is better throughout the course of the treatment. The hint will affect the clinical effects and bring interference to the study results.

### 3. Reviewing sites' CRFs for completeness and internal consistency

In order to affirm the validity, integrity, and accuracy of research documents, monitors have to look up all of the CRFs one by one. This step is not only the focus of monitoring, but also a controversy issue. We have learned about experiences from domestic and international counterparts in the pharmaceutical clinical research [13-15].

#### (1) Verifying the patients' eligibility and validity

Monitors verify the informed consent of autograph, and randomly call the patients with the contact phone number provided on the CRF, so as to ensure the authenticity of the subjects, and to check the diagnosis and the main treatment experiences. At the same time, monitors review the original medical histories and laboratory tests (e.g. routine test of blood, urine and stool, electrocardiogram, liver function, and kidney function) in order to affirm the patients eligibility.

#### (2) Data monitoring

Monitors ensure the data recording in CRFs are compliant with the medical records and laboratory reports. In particular, the monitors need to review records for key elements and data validation. The following aspects are practiced:

1) We check the testing time, the contents of enrolling laboratory tests, and the filling time in forms to guarantee the timeliness and integrity. Additionally, we make sure that the revision mistake must conform to the standard and has the signature of superintendent. We also review whether there is any alteration that cannot be explained by reasonable causes or intentional revision in the scales and the main indexes.

2) Some randomly selected subjects were asked on-the-spot regarding their case histories, treatments and drug combination in order to audit the facticity and completeness of CRF documents. The monitors specifically inquire treating histories including the information about the drug usage before patients' enrollment and whether patients combine the medication therapy during the research or even take the unapproved concomitant therapy in protocol. Moreover, if the patient took any drugs, the clinicians should record the information in the CRF form with the start-stops date. However, if the patients took the medication that is not allowed, we will evaluate the elimination if it is fit for the GCP principle. For instance, if the migraine patients took the medicine, we would ask the patients themselves and the doctors to truthfully document the name, the dosage of medicine, the time of taking the medicine, the time of pain relief and the side effects of the medicine. Furthermore, if a patient has taken prophylactic drugs to prevent migraine during the treatment period, the patient will be told to drop out according to the GCP and protocol.

3) We supervise assessment for adverse events. There are some arguments on acupuncture-induced adverse events in the international acupuncture academia at present, and the domestic clinical trials of acupuncture on this issue were not fully reported previously. Therefore, sponsors and monitors pay special attention on the recording and verification of safety monitoring and adverse events in acupuncture studies. Adverse events are classified by the State Food and Drug Administration (SFDA) of China using two criteria in specific order: adverse event and serious adverse event. Serious adverse events refer to required hospitalization, extended duration of hospitalization, disability, impact on the ability to work, life-threaten or death, resulting in events such as congenital malformations in the process of clinical trials. Up to now, the monitors have confirmed that there is no case of serious adverse events in all of these trials. Any adverse events must be recorded, including serious bleeding, hematoma, fainting, serious pain and local infection. How to deal with these events must be recorded as well. All of the adverse events must be handled and followed up. Take a case for example: a patient (random number:216) had subcutaneous hematoma and ecchymosis at the point of "Zusanli" (ST36) which appeared around one centimeter in diameter after the needle was withdrew. The physician gave local cold compress immediately, and advised the patient to hot compress after 24 hours. The patient's hematoma problem improved significantly the next day and all the symptoms completely disappeared after two weeks.

4) We audit the promptness and completeness of follow-up. All the patients in these trials need to complete the outcome measurements after treatment in 4 and 12 weeks, in order to assess and compare the long-term follow-up results in different treatment group. But not everyone has enough time to return visit to receive the scale evaluation actually. As a result, monitors have to inspect assessors to document whether or not the way of follow-up, including by out-patient, telephone, email, and webcam. During the follow-up, Bell's palsy patients mostly selected out-patient or webcam because they cannot judge the function of facial muscle by themselves. On the contrary, migraine patients eagerly used telephone or email to complete the scale evaluation.

#### (3) Verifying the description of subjects dropout and lost to follow-up

Monitors have accountability to know the quantities of dropouts, suspension and lost to follow-up, and assess the authenticity and comprehensiveness of reasons. We check if the details of withdraw in CRFs have been recorded. Generally speaking, the causes of withdraw commonly have the following conditions: Firstly, the subjects are too busy to complete the treatment course. Secondly, patients return to home town and they cannot stick to the treatment. Thirdly, patients violate advice and refuse to comply with the protocol.

If these kinds of subjects constituted a large proportion, doctors should arouse patients' initiative, improve the attitude in clinics, and exhibit more concerns in order to obtain patients' trust and cooperation, and strengthen their compliance.

#### (4) Evaluating the conservation of materials

Each clinical center should keep the entire files well, including patients' records, laboratory checking reports, CRF forms regardless of their filing status, and other essential documents. Monitors evaluate whether there is omission or loss in relevant materials and reports.

### Final report on quality monitoring

After assessing of on-site performance, monitors have to accomplish the monitoring report. The report is submitted to QA and the sponsors. All reports must be completed in handwriting. The monitors put forward recommendations and suggestions to every cooperative center. The contents of monitoring report should focus on review of regulatory documents, acupuncture audit, recommendations for improving performance, and the rectification on the previous monitors' recommendations. By holding regular meetings to discuss improvements in monitoring standards, the monitors can improve quality and efficiency.

## Summary and Conclusions

The National Basic Research Program (*973 Program*) and the "11^th ^Five-year Plan" National Key Technology R & D program are the most important research program based on clinical practice in China. Up to now, these projects are respectively the largest clinical trial involved in the specific physiological effects of acupoints[13], and one of the largest randomized controlled trials addressing the effectiveness of acupuncture treatment, and the best acupuncture treatment method for Bell's palsy[16] Monitoring work in these multi-centre large sample acupuncture clinical trials are as serious and scientific as pharmaceutical clinical test, which is essential for data accuracy and proper evaluation of study objectives in clinical trials.

Monitors should look up all of the source documents and source data, and assess whether the coordinators carry out these researches under the requirement of GCP with an objective, rigorous, careful, and scientific attitude.

Monitoring provides clinicians and assessors with the opportunity to discover problems early enough so that they can be resolved. If data quality drops at the end of the study, the results will have little value beyond providing a cautionary example for future studies. Through the review once a month at least, we can assist with corrective action as needed. Points for attention during the monitoring processes:

### (1) Rechecking abnormal laboratory indicators as necessary as possible

When subjects enrolled, few indexes in laboratory checking are unusual, such as the bradycardia in ECG test or low leukocyte, but they did not have any correlated history or pathological symptoms. The doctor should ask the subjects to recheck these indicators in 3 days to confirm that the performance for pathological or physiological abnormality, and provide specialist consultation when necessary. The patients will do the same examination after they finish the treatment to clear whether acupuncture has harmful effect.

### (2) Inquiring treatment combination initiatively

During the period of therapy, patients might be affected by their relatives or friends, go for other treatments other than the prescribed medications. Most importantly, they do not actively inform the clinicians about their additional medication. Therefore, during the treatment process, it is necessary for the clinicians to enhance communication with patients and often inquire the concomitant therapy comprehensively during the treatment process.

### (3) Supervising the qualified researchers in trials

During the implementation of clinical trials, some senior postgraduate students will graduate sooner or later, so every successor who will take part in the research should pass the relevant training and examinations, in the hope that they will master basic knowledge of GCP and completely control the implementation of research processes, and qualify themselves for the trials.

Monitors' responsibilities are not only the clinical inspection, but facilitating collaboration among study coordinators and sponsors.

1) During the audit schedule, we try to know the practical difficulties and opinions in clinical centers through informal discussions, and we make efforts to resolve such difficulties, if necessary, through the sponsor to coordinate.

2) We can find out the problems in each unit existing in the course of implementation through consulting the sub-centers monitoring reports, and propose the solutions other coordinators gained as reference.

3) The large sample, multi-center clinical trials have certain characteristics: Firstly, it has high-quality requirements in inclusion criteria. Secondly, the treating course and follow-up period is relatively long. Thirdly, the mission is arduous, and clinicians have to pay much attention to details. The investigators tend to get tired and volatile, and this downside leads to a negative effect towards the quality when the clinicians face the difficulties. The monitors should encourage them, give them timely information regarding the progress of the project, and let them be aware of the importance and necessity of their work. The monitors can pave the way for enhancing the coordination's exchange experience, for making good play of the trial.

In summary, the quality assurance is very important in acupuncture clinical research. Supplementing and improving the existed guidelines for quality monitoring will ensure that large multi-centre acupuncture clinical trials will be considered as valid and scientifically stringent as pharmaceutical clinical trials. It will also develop academic excellence and further promote the international recognition of acupuncture.

## Competing interests

The authors declare that they have no competing interests.

## Authors' contributions

LZ drafted the manuscript. All authors contributed to the further writing of the manuscript as well as read and approved the final manuscript.

## Supplementary Material

Additional file 1 **Appendix**. Monitoring record. Click here for file
