# Supplementary material for: Improved quality monitoring of multi-center acupuncture clinical trials in China
Source: Trials. 2009 Dec 27;10:123. doi: 10.1186/1745-6215-10-123 (PMC2806366; doi:10.1186/1745-6215-10-123)
Supplement: Additional file 1 — Appendix. Monitoring record. [file 1745-6215-10-123-S1.DOC]

**Appendix:**

Monitoring Record

| **The Hospital Name:** | | |  | | | | | | | |
| --- | --- | --- | --- | --- | --- | --- | --- | --- | --- | --- |
| **Monitor Name:** | | |  | | | | | | | |
| **The Organization of monitor:** | | |  | | | | | | | |
| **Audit Date:** | | |  | | | | | | | |
| **Submit Date:** | | |  | | | | | | | |
| **1. Communication objects** | | | | | | | | | | |
| **Name** | **Status** | | | | | | | | | |
|  | **Director of clinical center** | | | **Director of hospital** | **Patient** | | **clinician** | | **Laboratory Technician** | |
|  | □ | | | □ | □ | | □ | | □ | |
|  | □ | | | □ | □ | | □ | | □ | |
|  | □ | | | □ | □ | | □ | | □ | |
|  | □ | | | □ | □ | | □ | | □ | |
|  | □ | | | □ | □ | | □ | | □ | |
|  | □ | | | □ | □ | | □ | | □ | |
|  | □ | | | □ | □ | | □ | | □ | |
| **2. Subject selected** | |  | | | | | | | | |
| **Items** | | **Number** | | | | | | | | |
| Waiting list | |  | | | | | | | | |
| Screening period | |  | | | | | | | | |
| Reject after enrollment | |  | | | | | | | | |
| Enter the treatment period | |  | | | | | | | | |
| In the period of treatment | |  | | | | | | | | |
| Drop-off during treatment | |  | | | | | | | | |
| Complete treatment | |  | | | | | | | | |
| Enter the follow-up period | |  | | | | | | | | |
| In the period of follow-up | |  | | | | | | | | |
| Complete follow-up | |  | | | | | | | | |
| **3. Subject drop-off** | |  | | | | | | | | |
| **The reason for drop-off and the random number** | | | | | | **Number** | | | | |
|  | | | | | |  | | | | |
|  | | | | | |  | | | | |
|  | | | | | |  | | | | |
|  | | | | | |  | | | | |
|  | | | | | |  | | | | |
|  | | | | | |  | | | | |
|  | | | | | |  | | | | |
|  | | | | | |  | | | | |
|  | | | | | |  | | | | |
|  | | | | | |  | | | | |
| **4. On-site inspection records** | | | | | | | |  | | |
|  |  |  |  |  |  |  |  | **Yes** | | **No** |
| **(1)Authenticity of the informed consent** | | | | | | | | □ | | □ |
| **(2)Protocol adherence** | | | | | | | | □ | | □ |
| Meets the criteria for diagnosis | | | | | | | | □ | | □ |
| Meets the criteria for inclusion/exclusion criteria | | | | | | | | □ | | □ |
| Laboratory tests on schedule | | | | | | | | □ | | □ |
| Perform the treatment according to random scheme | | | | | | | | □ | | □ |
| Perform the therapy according to SOP | | | | | | | | □ | | □ |
| Unapproved/unauthorized concomitant therapy used | | | | | | | | □ | | □ |
| **(3)Safety monitoring** | | | | | | | | □ | | □ |
| Record adverse events on CRF authentically | | | | | | | | □ | | □ |
| Report serious adverse events on CRF authentically and submit to sponsor | | | | | | | | □ | | □ |
| Follow-up adverse events | | | | | | | | □ | | □ |
| **(4)Essential documents** | | | | | | | | □ | | □ |
| Integrity of case histories records | | | | | | | | □ | | □ |
| Complete CRF timely | | | | | | | | □ | | □ |
| The validity, integrity, and accuracy of CRF | | | | | | | | □ | | □ |
| Data found in source documents are recorded on CRF as required | | | | | | | | □ | | □ |
| Data in CRF are in accord with electro-CRF | | | | | | | | □ | | □ |
| Data correction conform to the standard | | | | | | | | □ | | □ |
| Record dropouts and reasons in detail | | | | | | | | □ | | □ |
| Record the concomitant therapy used | | | | | | | | □ | | □ |

**5. Summary assessment**

**6. Pictures records**
